# A Novel Tool to Predict Early Death in Uterine Sarcoma Patients: A Surveillance, Epidemiology, and End Results-Based Study

**DOI:** 10.3389/fonc.2020.608548

**Published:** 2020-11-26

**Authors:** Zixuan Song, Yizi Wang, Dandan Zhang, Yangzi Zhou

**Affiliations:** Department of Obstetrics and Gynecology, Shengjing Hospital of China Medical University, Shenyang, China

**Keywords:** uterine sarcoma, early death, nomograms, Surveillance, Epidemiology, and EndResults database, prognosis

## Abstract

**Background:**

Uterine sarcoma is a rare gynecologic tumor with a high degree of malignancy. There is a lack of effective prognostic tools to predict early death of uterine sarcoma.

**Methods:**

Data on patients with uterine sarcoma registered between 2004 and 2015 were extracted from the Surveillance, Epidemiology, and End Results (SEER) data. Important independent prognostic factors were identified by univariate and multivariate logistic regression analyses to construct a nomogram for total early deaths and cancer-specific early deaths.

**Results:**

A total of 5,274 patients with uterine sarcoma were included in this study. Of which, 397 patients experienced early death (≤3 months), and 356 of whom died from cancer-specific causes. A nomogram for total early deaths and cancer-specific early deaths was created using data on age, race, tumor size, the International Federation of Gynecology and Obstetrics (FIGO) staging, histological classification, histological staging, treatment (surgery, radiotherapy, chemotherapy), and brain metastases. On comparing the C-index, area under the curve, and decision curve analysis, the created nomogram showed better predictive power and clinical practicality than one made exclusively with FIGO staging. Calibration of the nomogram by internal validation showed good consistency between the predicted and actual early death.

**Conclusions:**

Nomograms that include clinical characteristics can provide a better prediction of the risk of early death for uterine sarcoma patients than nomograms only comprising the FIGO stage system. In doing so, this tool can help in identifying patients at high risk for early death because of uterine sarcoma.

## Introduction

Uterine sarcoma is a rare gynecologic malignancy with poor prognosis and comprises approximately 1% of female genital malignancies and 3%–7% of uterine tumors (1, 2). It is mainly divided into two categories: a) mesenchymal tumors and b) mixed epithelial and mesenchymal tumors. The former can be further classified as endometrial stromal sarcomas, leiomyosarcomas, and miscellaneous tumors. Adenosarcoma, malignant mixed Müllerian tumor (MMMT), and carcinosarcoma are considered mixed epithelial and mesenchymal tumors. Although carcinosarcoma is classified as a uterine sarcoma according to the World Health Organization, it is currently considered an endometrial cancer (3). Uterine sarcoma has a poor prognosis, and existing common treatments, such as surgery, radiotherapy, and chemotherapy, are not effective (4). The 5-year survival rate is 50%–55% for patients with early uterine sarcoma and 8%–12% for advanced cases (5, 6). However, few studies have examined patients who died early. Early detection of patients at a high risk of death may help design personalized treatment regimens that improve the patients’ survival and quality of life. In a study of advanced soft tissue sarcomas, the authors performed a retrospective analysis and established a prognostic model of early death within 3 months (7). This study provides us with insights into early mortality. At present, no study thoroughly investigates the 3-month mortality rate of uterine sarcoma patients, therefore, warranting the need for a predictive model for the early death in these patients.

The International Federation of Gynecology and Obstetrics (FIGO) staging system is commonly used for gynecological tumors. The latest staging for uterine sarcoma is found in FIGO 2009 (8). Patients with uterine sarcoma are staged as per the degree of tumor invasion, regional lymph node positivity, and presence of distant metastasis; the higher the stage, the worse the prognosis. However, the FIGO staging does not account for pathologic grade; age, race, and other related factors; or the prognosis of uterine sarcoma related to treatment. Therefore, there are limitations in predicting early death of uterine sarcoma based on the FIGO staging.

Further, the Linked Surveillance, Epidemiology, and End Results (SEER) database (https://seer.cancer.gov/) is an authoritative source of information concerning cancer incidence and survival in the United States. Currently, tumor incidence and survival data of approximately 34.6% of the US population cancer registry are collected and published in it. Our study assessed the incidence of early death in patients with uterine sarcoma registered in the SEER database between 2004 and 2015 and aimed to create a predictive model that overcomes the limitations of models based exclusively on the FIGO staging.

## Materials and Methods

### Patients

**Figure 1:**
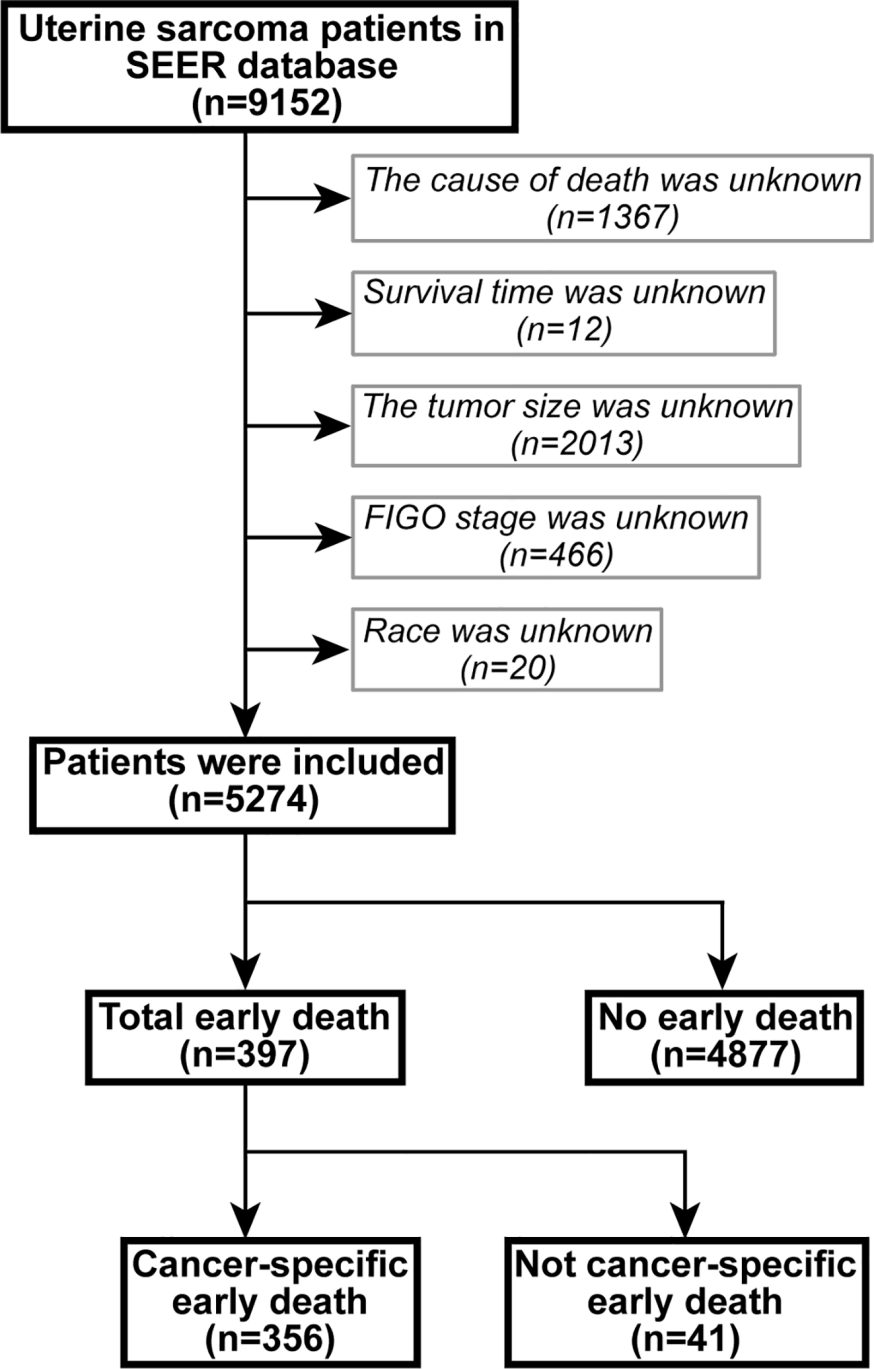
The flowchart of patient selection.

The data were extracted using SEER*Stat version 8.3.6.1. In the SEER database of patients with uterine sarcoma, registered from 2004 to 2015 according to the International Classification of Tumor Diseases Third Edition (ICD-O-3), the included site codes were c54.0–C54.3, C54.8, and C54.9; these were histologically coded as 8800/3–8950/3, 8963/3, and 8982/3-8991/3, respectively. Carcinosarcoma was not included in this study because it was classified as endometrial cancer (1). Exclusion criteria were: 1) unknown cause of death, 2) unknown survival period, 3) unknown tumor size, 4) undetermined FIGO staging, and 5) unknown race. A flowchart of patient selection criteria is shown in **Figure 1**.

### Data Collection

**Figure 2:**
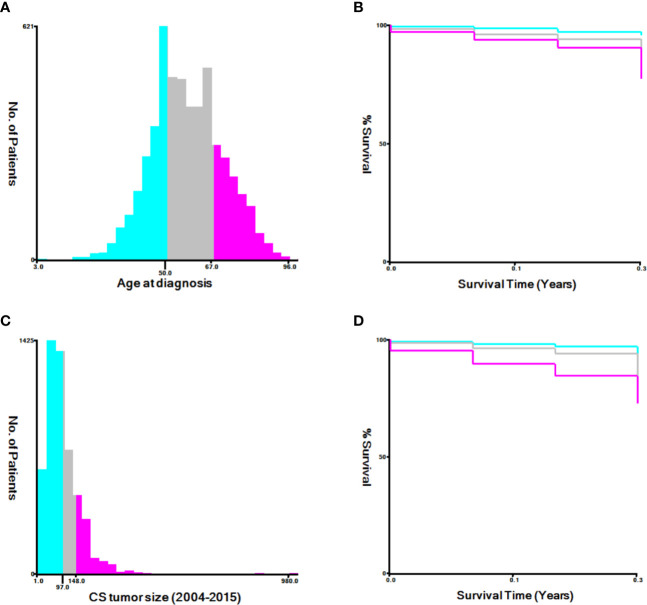
The appropriate cutoff values of age and tumor size was assessed by X-tile analysis. **(A, B)** The appropriate cutoff values of age were 50 and 67 years; **(C, D)** The appropriate cutoff values of tumor size were 97 and 148 mm.

Demographic and clinical characteristics of the patients were extracted from the SEER database, including age at diagnosis, race, tumor size, histological grade, FIGO staging, treatment (surgery, radiotherapy, and chemotherapy), and metastases (bone, brain, liver, and lung). Appropriate cutoff values for age and tumor size were assessed using the X-tile software (9) (**Figure 2**) and determined as 50 and 67 years and 97 and 148 mm, respectively. The primary outcome of this study was all-cause and cancer-specific early death. Early death was defined as a survival time ≤3 months after the initial diagnosis of uterine sarcoma (7).

### Statistical Analysis

The X-tile software was used to assess the optimal cutoff values for age and tumor size. Rsize. R Version 4.0.2 (R Foundation for Statistical Computing, Vienna, Austria, http://www.r-project.org) was used to analyze all data in the RStudio environment. P<0.05 was considered statistically significant. Univariate and multivariate logistic regression analyses were performed using the clinical data to assess the factors associated with early mortality. Odds ratios (ORs) and 95% confidence intervals (CIs) were calculated. Nomograms were constructed to predict early death in patients with uterine sarcoma based on associated risk factors. The calibration of nomograms was evaluated by generating a calibration diagram by bootstrapping (1,000 resamplings). Nomograms were evaluated by studying the area under the curve (AUC) of the receiver operating characteristic curve (ROC) (10). The concordance statistic (C-statistic) (11) and Brier score (12) of the original model and the verified model were compared through internal validation by bootstrapping (1000 resamplings). The clinical effects of the nomogram were evaluated by a decision curve analysis (DCA) (13) that calculated the net gain at each risk threshold probability.

## Results

### Patient Characteristics

There were 9,152 patients with uterine sarcoma in the SEER database, of which 5,274 patients were included in the study based on the inclusion and exclusion criteria. Among them, there were 397 patients who died early, and 356 who died because of cancer-specific causes. Most of those who died early were white (66%) and aged between 51 and 67 years (46%). The most common pathologic types were leiomyosarcoma (33%), Müllerian mixed tumor (33%), FIGO IV (68%), and GIII–GIV (62%). The majority of patients who died early had no bone (55%), brain (58%), liver (52%), and/or lung metastases (40%). Most early deaths were treated surgically (72%), without radiotherapy (92%) and without chemotherapy (74%). The characteristics of the patients who did or did not experience early death are shown in **Table 1**.

**Table 1:** Characteristics with uterine sarcoma patients.

Characteristic	No early death No. (%)	Total early death No. (%)	Cancer-specific early death No. (%)
**Total**	4877	397	365
**Age**			
≤50	1,525 (31%)	58 (15%)	52 (15%)
51–67	2,253 (46%)	184 (46%)	166 (47%)
≥68	1,099 (23%)	155 (39%)	138 (39%)
**Race**			
White patients	3,476 (71%)	263 (66%)	236 (66%)
African American	935 (19%)	114 (29%)	101 (28%)
Others	466 (9.6%)	20 (5.0%)	19 (5.3%)
**Tumor size(mm)**			
≤97	3,056 (63%)	132 (33%)	115 (32%)
98–148	1,070 (22%)	98 (25%)	92 (26%)
≥149	751 (15%)	167 (42%)	149 (42%)
**FIGO Stage**			
FIGO Stage I	2,926 (60%)	31 (7.8%)	19 (5.3%)
FIGO Stage II	385 (7.9%)	15 (3.8%)	13 (3.7%)
FIGO Stage III	680 (14%)	82 (21%)	79 (22%)
FIGO Stage IV	886 (18%)	269 (68%)	245 (69%)
**Pathological classification**			
Leiomyosarcoma	2,128 (44%)	132 (33%)	121 (34%)
Endometrial stromal sarcoma	883 (18%)	71 (18%)	70 (20%)
Mixed epithelial and mesenchymal tumors	1,689 (35%)	130 (33%)	110 (31%)
Others	177 (3.6%)	64 (16%)	55 (15%)
**Histology grade**			
GI–GII	933 (19%)	10 (2.5%)	9 (2.5%)
GIII–GIV	2,322 (48%)	246 (62%)	220 (62%)
Unknown	1,622 (33%)	141 (36%)	127 (36%)
**Surgery**			
No	111 (2.3%)	109 (27%)	97 (27%)
Yes	4,765 (98%)	286 (72%)	257 (72%)
Unknown	1 (<0.1%)	2 (0.5%)	2 (0.6%)
**Radiation**			
No	3,636 (75%)	367 (92%)	328 (92%)
Yes	1,241 (25%)	30 (7.6%)	28 (7.9%)
**Chemotherapy**			
No	2,856 (59%)	294 (74%)	261 (73%)
Yes	2,021 (41%)	103 (26%)	95 (27%)
**Bone-metastases**			
No	2,641 (54%)	220 (55%)	196 (55%)
Yes	50 (1.0%)	16 (4.0%)	16 (4.5%)
Unknown	2,186 (45%)	161 (41%)	144 (40%)
**Brain-metastases**			
No	2,686 (55%)	230 (58%)	206 (58%)
Yes	5 (0.1%)	5 (1.3%)	5 (1.4%)
Unknown	2,186 (45%)	162 (41%)	145 (41%)
**Liver-metastases**			
No	2,627 (54%)	205 (52%)	184 (52%)
Yes	64 (1.3%)	32 (8.1%)	29 (8.1%)
Unknown	2,186 (45%)	160 (40%)	143 (40%)
**Lung-metastases**			
No	2,442 (50%)	157 (40%)	140 (39%)
Yes	245 (5.0%)	79 (20%)	72 (20%)
Unknown	2,190 (45%)	161 (41%)	144 (40%)

FIGO, Federation International of Gynecology and Obstetrics.

### Risk Factor Analysis for Early Death

Univariate and multivariate logistic regressions for total early death and cancer-specific early death are shown in **Tables 2** and **3**. Univariate analysis showed that, in general, early death and cancer-specific mortality was higher among black patients; those who were older; had larger tumors, higher FIGO staging, higher histological grade of tumors, and other types of uterine sarcomas(except for leiomyosarcoma, endometrial stromal sarcoma and mixed epithelial and mesenchymal tumors); did not undergo surgery, radiotherapy, or chemotherapy; or had bone, brain, liver, or lung metastasis. Multivariate analysis results showed that, in general, early death and cancer-specific mortality risk was higher among black patients; older patients (51–67 years old, at the age of 68 or higher); those with larger tumors (98–148 mm, 149 mm or higher), higher FIGO staging (II, III, IV), or higher histological grade (GIII–GIV); those with tumors like endometrial stromal sarcoma, mixed epithelial and mesenchymal tumors, or other types of uterine sarcomas(except for leiomyosarcoma, endometrial stromal sarcoma and mixed epithelial and mesenchymal tumors); those who did not undergo surgery, radiotherapy, or chemotherapy; and those with brain metastases.

**Table 2:** The univariable and multivariate logistic regression analysis of total early death.

Characteristic	Univariate logistic Regression			Multivariate logistic Regression		
	OR	95% CI	P-value	OR	95% CI	P-value
**Age**						
≤50	Ref			Ref		
51–67	2.15	1.60, 2.93	<0.001*	1.67	1.17, 2.41	0.006*
≥68	3.71	2.73, 5.10	<0.001*	2.75	1.86, 4.09	<0.001*
**Race**						
White patients	Ref			Ref		
African American	1.61	1.27, 2.03	<0.001*	1.47	1.10, 1.96	0.009*
Others	0.57	0.35, 0.88	0.017*	0.71	0.40, 1.21	0.228
**Tumor size(mm)**						
≤97	Ref			Ref		
98–148	2.12	1.61, 2.78	<0.001*	1.44	1.03, 2.01	0.031*
≥149	5.15	4.05, 6.56	<0.001*	2.39	1.74, 3.28	<0.001*
**FIGO Stage**						
FIGO Stage I	Ref			Ref		
FIGO Stage II	3.68	1.92, 6.76	<0.001*	3.16	1.59, 6.02	<0.001*
FIGO Stage III	11.4	7.55, 17.6	<0.001*	9.86	6.33, 15.7	<0.001*
FIGO Stage IV	28.7	19.9, 42.7	<0.001*	25.6	16.8, 40.0	<0.001*
**Pathological classification**						
Leiomyosarcoma	Ref			Ref		
Endometrial stromal sarcoma	1.30	0.96, 1.74	0.089	2.82	1.90, 4.17	<0.001*
Mixed epithelial and mesenchymal tumors	1.24	0.97, 1.59	0.091	1.75	1.26, 2.44	<0.001*
Others	5.83	4.15, 8.13	<0.001*	4.4	2.77, 6.95	<0.001*
**Histology grade**						
GI–GII	Ref			Ref		
GIII–GIV	9.88	5.52, 20.0	<0.001*	7.7	3.98, 16.6	<0.001*
Unknown	8.11	4.48, 16.5	<0.001*	6.69	3.37, 14.7	<0.001*
**Surgery**						
No	Ref			Ref		
Yes	0.06	0.05, 0.08	<0.001*	0.25	0.17, 0.36	<0.001*
Unknown	2.04	0.19, 44.2	0.564	2.5	0.21, 58.7	0.480
**Radiation**						
No	Ref			Ref		
Yes	0.24	0.16, 0.34	<0.001*	0.35	0.22, 0.53	<0.001*
**Chemotherapy**						
No	Ref			Ref		
Yes	0.50	0.39, 0.62	<0.001*	0.17	0.13, 0.23	<0.001*
**Bone-metastases**						
No	Ref			Ref		
Yes	3.84	2.09, 6.71	<0.001*	0.94	0.44, 1.91	0.862
Unknown	0.88	0.72, 1.09	0.253	0.66	0.01, 17.5	0.821
**Brain-metastases**						
No	Ref			Ref		
Yes	11.7	3.23, 42.3	<0.001*	8.07	1.58, 41.6	0.012*
Unknown	0.87	0.70, 1.07	0.175	3.99	0.19, 131	0.385
**Liver-metastases**						
No	Ref			Ref		
Yes	6.41	4.05, 9.95	<0.001*	1.7	0.94, 3.03	0.075
Unknown	0.94	0.76, 1.16	0.558	0.81	0.08, 7.71	0.849
**Lung-metastases**						
No	Ref			Ref		
Yes	5.02	3.70, 6.76	<0.001*	0.99	0.65, 1.51	0.957
Unknown	1.14	0.91, 1.44	0.248	0.44	0.07, 2.20	0.339

OR, odds ratio; CI, confidence interval; Ref, reference; FIGO, Federation International of Gynecology and Obstetrics.

* means p <0.05.

**Table 3:** The univariable and multivariate logistic regression analysis of cancer-specific early death.

Characteristic	Univariate logistic Regression			Multivariate logistic Regression		
	OR	95% CI	P-value	OR	95% CI	P-value
**Age**						
≤50	Ref			Ref		
51–67	2.16	1.58, 3.00	<0.001*	1.67	1.15, 2.46	0.008*
≥68	3.68	2.67, 5.15	<0.001*	2.92	1.94, 4.45	<0.001*
**Race**						
White patients	Ref			Ref		
African American	1.59	1.24, 2.02	<0.001*	1.44	1.06, 1.94	0.019*
Others	0.60	0.36, 0.94	0.036*	0.74	0.41, 1.29	0.310
**Tumor size(mm)**						
≤97	Ref			Ref		
98–148	2.28	1.72, 3.03	<0.001*	1.49	1.05, 2.11	0.023*
≥149	5.27	4.09, 6.82	<0.001*	2.36	1.69, 3.30	<0.001*
**FIGO Stage**						
FIGO Stage I	Ref			Ref		
FIGO Stage II	5.20	2.49, 10.5	<0.001*	4.62	2.15, 9.63	<0.001*
FIGO Stage III	17.9	11.0, 30.6	<0.001*	15.8	9.44, 27.6	<0.001*
FIGO Stage IV	42.6	27.3, 70.6	<0.001*	37.9	23.0, 65.4	<0.001*
**Pathological classification**						
Leiomyosarcoma	Ref			Ref		
Endometrial stromal sarcoma	1.39	1.02, 1.88	0.032*	3.05	2.03, 4.57	<0.001*
Mixed epithelial and mesenchymal tumors	1.15	0.88, 1.49	0.317	1.52	1.07, 2.16	0.019*
Others	5.46	3.82, 7.75	<0.001*	4.02	2.47, 6.49	<0.001*
**Histology grade**						
GI–GII	Ref			Ref		
GIII-GIV	9.82	5.33, 20.7	<0.001*	7.96	3.98, 17.9	<0.001*
Unknown	8.12	4.35, 17.3	<0.001*	7.04	3.42, 16.2	<0.001*
**Surgery**						
No	Ref			Ref		
Yes	0.06	0.05, 0.08	<0.001*	0.25	0.17, 0.38	<0.001*
Unknown	2.29	0.22, 49.7	0.502	2.88	0.24, 67.6	0.414
**Radiation**						
No	Ref			Ref		
Yes	0.25	0.17, 0.36	<0.001*	0.37	0.23, 0.57	<0.001*
**Chemotherapy**						
No	Ref			Ref		
Yes	0.51	0.40, 0.65	<0.001*	0.18	0.13, 0.24	<0.001*
**Bone-metastases**						
No	Ref			Ref		
Yes	4.31	2.34, 7.55	<0.001*	1.01	0.47, 2.07	0.972
Unknown	0.89	0.71, 1.11	0.294	0.56	0.01, 17.0	0.767
**Brain-metastases**						
No	Ref			Ref		
Yes	13.0	3.60, 47.2	<0.001*	8.44	1.66, 43.2	0.010*
Unknown	0.86	0.69, 1.08	0.196	4.38	0.19, 153	0.371
**Liver-metastases**						
No	Ref			Ref		
Yes	6.47	4.02, 10.2	<0.001*	1.7	0.93, 3.08	0.081
Unknown	0.93	0.74, 1.17	0.553	0.81	0.08, 7.54	0.848
**Lung-metastases**						
No	Ref			Ref		
Yes	5.13	3.73, 6.99	<0.001*	0.98	0.63, 1.52	0.939
Unknown	1.15	0.90, 1.46	0.262	0.47	0.07, 2.39	0.391

OR, Odds Ratio; CI, Confidence Interval; Ref, Reference; FIGO, Federation International of Gynecology and Obstetrics.

* means p <0.05.

### Nomogram Construction

**Figure 3:**
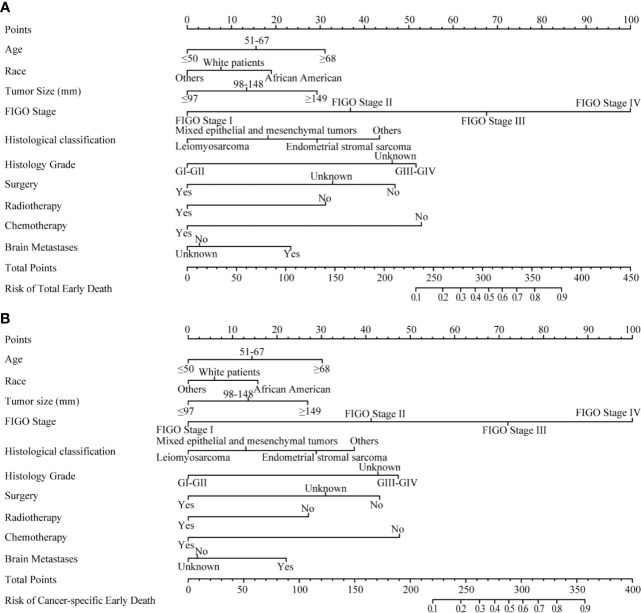
The nomograms of early death in patients with uterine sarcoma. **(A)** The total early death; **(B)** The cancer-specific early death.

Important variables in multivariate logistic regression, including age, race, tumor size, FIGO stage, pathological type, histological grade, treatment (surgery, radiotherapy, chemotherapy) and brain metastases, were selected to construct nomograms for total early death and cancer-specific early death (**Figure 3**). We also constructed nomograms to predict this outcomes based exclusively on the FIGO stages (**Supplementary Figure 1**).

### Performance of Nomograms

**Figure 4:**
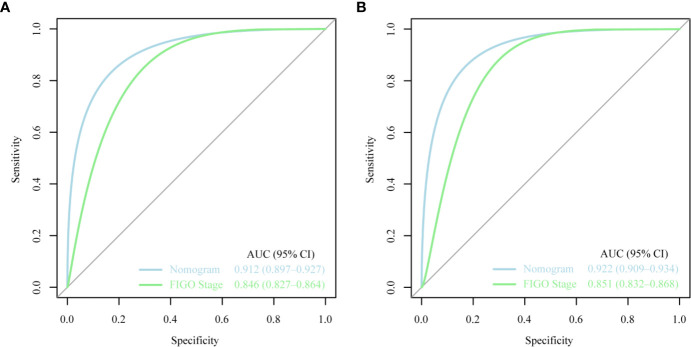
The receiver operating characteristic (ROC) curve for nomogram. **(A)** The total early death; **(B)** The cancer-specific early death. AUC, area under the curve; ROC, receiver operating characteristic.

**Figure 5:**
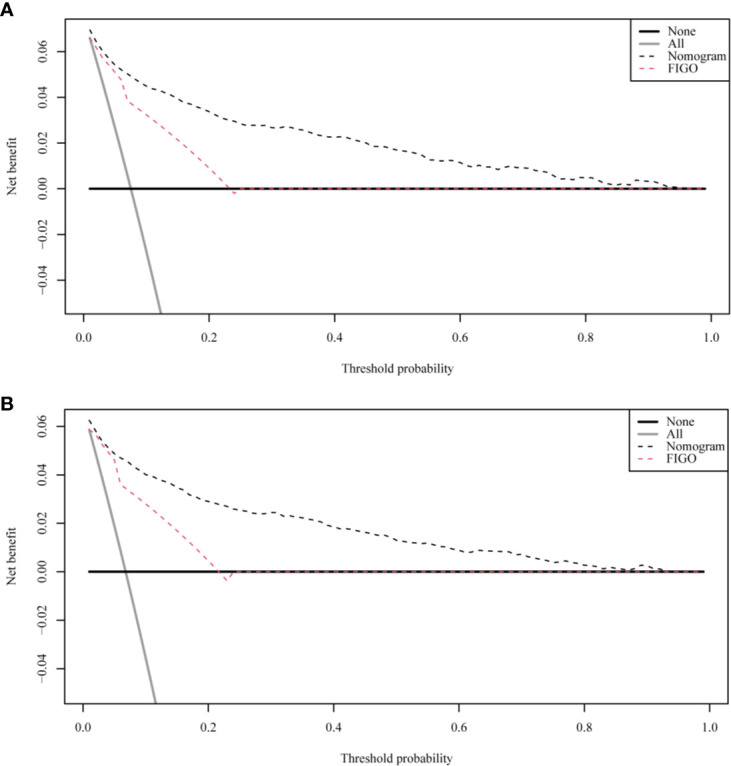
The decision curve analysis (DCA) curve for nomogram. **(A)** The total early death; **(B)** The cancer-specific early death.

**Figure 6:**
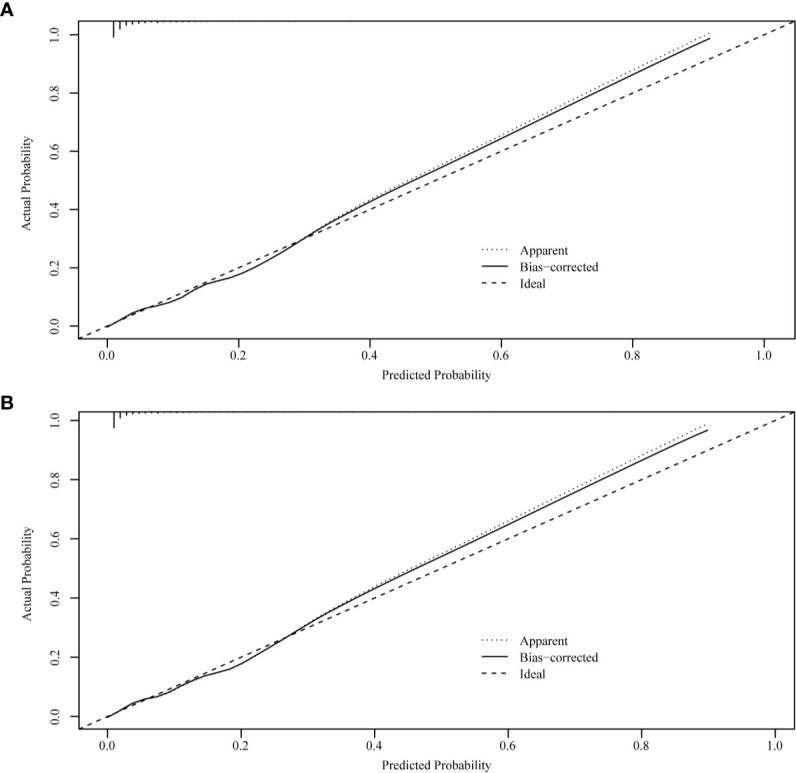
Internal verification plots of nomogram calibration curves by bootstrapping with 1,000 resamples. **(A)** The total early death; **(B)** The cancer-specific early death.

The ROC curves used to assess the nomograms of total early death and cancer-specific early death is shown in **Figure 4**. The AUC of the nomogram constructed using the variables selected from the multivariate logistic regressions was higher than that of the nomogram based exclusively on the FIGO stages, suggesting that the former had better ability to predict the total early death and cancer-specific early death. Further, the DCA of the former (**Figure 5**) appears to have better clinical benefits than the FIGO-based nomogram. There was appropriate consistency between the observed and predicted probabilities of nomograms of total early death and cancer-specific early death, and all calibration curves were close to the 45-degree line (**Figure 6**). Internal verification was performed on the nomograms of total early death and cancer-specific early death. The C-statistic and Brier score before and after internal verification were shown in **Table 4**. The internal validation for both nomograms showed a good level of agreement on the prediction value.

**Table 4:** C-statistic and Breir score of nomograms for the uterine sarcoma.

Characteristics	Training cohort	After internal verification
**C-statistic**		
Total early death	0.9118	0.9066
Cancer-specific early death	0.9206	0.9156
**Brier score**		
Total early death	0.0456	0.0468
Cancer-specific early death	0.0420	0.0431

## Discussion

Attention to early death is critical to improve the survival of uterine sarcoma patients. Uterine sarcomas have a high early mortality rate; in this study, the early (≤3 months) mortality rate was 7.53%. Therefore, a set of tools for predicting early death in such cases needs to be developed. In this study, the FIGO stage was not the only predictive factor for early death in uterine sarcoma patients. Other social factors and treatment measures, such as older age, black race, large tumor size, other types of uterine sarcoma, higher histological grades, lack of treatment (surgery, radiotherapy, chemotherapy), and presence of brain metastasis, were also associated with higher early total mortality and uterine sarcoma-specific mortality. The FIGO staging system is a commonly used method in gynecology to evaluate the prognosis of uterine sarcoma, but its limitations make it unable to provide a personalized prognosis prediction. Clinically, it is used to conduct stratified analysis on the age of patients according to groups, such as those aged ≤50 years, 51–80 years, and >80 years. For tumor size, the FIGO stage for stage I uterine sarcoma is divided into stage IA and IB based on whether the tumor size is greater than 50 mm. However, this method is unable to distinguish the effect of varying age and tumor size on the early death of uterine sarcoma patients. Therefore, the X-tile software was selected in our study to determine the optimal cutoff point for these variables.

Nomograms are widely used to predict the patients’ risk of disease and prognosis of malignant tumors (14–16). In gynecologic oncology, recent studies have focused on the development of nomograms based on the SEER database. Studies based on the SEER database constitute a relatively large population than those based on a single center. Zhu et al. (17) developed and verified nomograms of total and cancer-specific survival to predict the survival of endometrial cancer patients. Predictive models was constructed based on tumor size, clinical grade, histological grade, age and other factors to predict 3-year and 5-year OS and CSS for endometrial cancer. Xie et al. (18) developed and validated a nomogram of total cervical cancer survival to predict the survival of cervical cancer patients, FIGO staging, age, race, tumor size, pathological classification, and other important factors were used to predict 3-year and 5-year OS of cervical cancer. However, there is currently no nomogram to predict early death from gynecologic tumors. Uterine sarcoma is a tumor with a high early mortality rate, in our study, the percentage of early deaths was as high as 7.5%. Therefore, an effective tool is needed to predict early death of uterine sarcoma to provide better personalized treatment that improves the quality of life in patients at high risk of early death. Clinically, gynecologic oncologists are used to use FIGO staging for uterine sarcoma prognostic analysis, ignoring other factors such as tumor size, histological grade, histological classification, age, and ethnicity. For rare tumors such as uterine sarcoma, the model constructed by the single-center study has poor predictive ability due to the small number of included cases. The SEER database was used in this study, which included not only a large number of cases of uterine sarcoma, but also a large amount of clinical information on patient age, race, tumor size, and so on. Therefore, our forecasting model contains more comprehensive forecasting factors and has stronger forecasting ability. The SEER Database-based Nomogram improves the ability to predict early death. The ROC curve and DCA (**Figure 4**) in our study show that nomograms created using patient-specific clinical characteristics could better predict the early death of uterine sarcoma patients and were more clinically practical than FIGO staging. By internal validation of the nomogram, the predicted early mortality rate and the actual early mortality rate were found to be consistent.

In recent years, many molecular markers for uterine sarcoma have been studied. Zhou JG et al. (6) constructed a predictive model containing FGF23, TLX2, TIFAB, RNF223, HIST1H3A and AADACL4 genes to predict the prognosis of uterine sarcoma. Compared with the patients in the low-risk group, the patients in the high-risk group showed significant mutation characteristics. Yokoi A et al. (19) constructed an effective diagnostic model for uterine leiomyosarcoma using seven serum miRNA (Mir-4430, Mir-6511b-5p, Mir-191-5p, Mir-451A, Mir-4485-5p, Mir-4635, and Mir-1246) with high diagnostic performance for preoperative screening of uterine sarcoma, and proved that serum miRNA could be used as a preoperative biomarker. Unfortunately, since the SEER database itself does not contain a large number of molecular markers, our study did not construct a model containing molecular markers.

The histological classification of uterine sarcomas also affects their prognosis. Lange SS et al. (20) showed that uterine leiomyosarcoma has a 5-year survival rate of 25%–75%, while endometrial stromal sarcoma is an indolent tumor with late local and distant recurrence. However, in recent years, endometrial stromal sarcoma was divided into high-grade endometrial stromal sarcoma and low-grade endometrial stromal sarcoma. High-grade endometrial stromal sarcoma recurrences usually occur earlier (<1 year), higher risk of death (21). Because the SEER database is limited, high-grade and low-grade endometrial stromal sarcomas cannot be completely distinguished, our study combined the two. In our study, endometrial stromal sarcoma was associated with a higher risk of early death than uterine leiomyosarcoma.

This model, however, also has some limitations. First, factors affecting early death that were not included in the SEER database were subsequently not included in the study. For example, although studies have shown that the survival rate of low-income patients with soft tissue sarcomas decreases regardless of disease stage, we could not evaluate the income parameter (22). Second, this was a retrospective study, and selection bias was inevitable. Third, the study did not consider specific surgical procedures, chemotherapy regimens, or radiotherapy regimens that may influence the prediction of early death. Fourth, the SEER database contains a wealth of unknown data on histological grade, bone metastases, brain metastases, liver metastases, and lung metastases, which can have an impact on predictive models. Fifth, more and more research is focused on the molecular mechanism of uterine sarcoma (23), since the SEER database does not contain molecular information for tumors, our model was unable to integrate the molecular information for uterine sarcomas. In addition, no external data were available to validate the model. In the future, the early death of uterine sarcoma patients needs to be predicted in combination with other research data.

In conclusion, the nomogram created in this study appears to be a better predictor of early death for uterine sarcoma patients than the one solely comprising the FIGO staging system does. Therefore, this nomogram can be used as a more effective prediction tool for uterine sarcoma cases in future clinical practice.

## Data Availability Statement

Publicly available datasets were analyzed in this study. This data can be found here: Surveillance, Epidemiology, and End Results (SEER) database (https://seer.cancer.gov/).

## Author Contributions

ZS: Research ideas, drafting drafts. YW: Data extraction and statistical analysis. DZ: Statistical analysis and manuscript writing. YZ: Conception of research, quality control. All authors contributed to the article and approved the submitted version.

## Conflict of Interest

The authors declare that the research was conducted in the absence of any commercial or financial relationships that could be construed as a potential conflict of interest.

## References

[1] Brooks SE Zhan M Cote T Baquet CR Surveillance, epidemiology, and end results analysis of 2677 cases of uterine sarcoma 1989-1999. Gynecol Oncol (2004) 93(1):204–8. 10.1016/j.ygyno.2003.12.029 15047237

[2] Major FJ Blessing JA Silverberg SG Morrow CP Creasman WT Currie JL Prognostic factors in early-stage uterine sarcoma. A Gynecologic Oncology Group study. Cancer (1993) 71(4 Suppl):1702–9. 10.1002/cncr.2820710440 8381710

[3] Kurman RJ Carcangiu ML Herrington CS Young RH WHO classification of tumours of female reproductive organs. 4th ed. Lyon: International Agency for Research on Cancer (2014).

[4] Rizzo A Pantaleo MA Saponara M Nannini M Current status of the adjuvant therapy in uterine sarcoma: A literature review. World J Clin Cases (2019) 7(14):1753–63. 10.12998/wjcc.v7.i14.1753 PMC6692269 31417921

[5] Fletcher CD The evolving classification of soft tissue tumours - an update based on the new 2013 WHO classification. Histopathology (2014) 64(1):2–11. 10.1111/his.12267 24164390

[6] Zhou JG Zhao HT Jin SH Tian X Ma H Identification of a RNA-seq-based signature to improve prognostics for uterine sarcoma. Gynecol Oncol (2019) 155(3):499–507. 10.1016/j.ygyno.2019.08.033 31662204

[7] Penel N Glabbeke MV Mathoulin-Pelissier S Judson I Sleijfer S Bui B Performance status is the most powerful risk factor for early death among patients with advanced soft tissue sarcoma: the European Organisation for Research and Treatment of Cancer-Soft Tissue and Bone Sarcoma Group (STBSG) and French Sarcoma Group (FSG) study. Br J Cancer (2011) 104(10):1544–50. 10.1038/bjc.2011.136 PMC3101912 21505457

[8] Prat J FIGO staging for uterine sarcomas. Int J Gynaecol Obstet (2009) 104(3):177–8. 10.1016/j.ijgo.2008.12.008 19135669

[9] Camp RL Dolled-Filhart M Rimm DL X-tile: a new bio-informatics tool for biomarker assessment and outcome-based cut-point optimization. Clin Cancer Res (2004) 10(21):7252–9. 10.1158/1078-0432.CCR-04-0713 15534099

[10] Janssens A Martens FK Reflection on modern methods: Revisiting the area under the ROC Curve. Int J Epidemiol (2020) 49(4):1397–403. 10.1093/ije/dyz274 31967640

[11] Pencina MJ D’Agostino RB Overall C as a measure of discrimination in survival analysis: model specific population value and confidence interval estimation. Stat Med (2004) 23(13):2109–23. 10.1002/sim.1802 15211606

[12] Roulston M Performance targets and the brier score. Meteorol Appl (2007) 14(2):185–94. 10.1002/met.21

[13] Van Calster B Wynants L Verbeek JFM Verbakel JY Christodoulou E Vickers AJ Reporting and Interpreting Decision Curve Analysis: A Guide for Investigators. Eur Urol (2018) 74(6):796–804. 10.1016/j.eururo.2018.08.038 30241973 PMC6261531

[14] Pabinger I van Es N Heinze G Posch F Riedl J Reitter EM A clinical prediction model for cancer-associated venous thromboembolism: a development and validation study in two independent prospective cohorts. Lancet Haematol (2018) 5(7):e289–e98. 10.1016/S2352-3026(18)30063-2 PMC7338218 29885940

[15] Wang X Mao M Xu G Lin F Sun P Baklaushev VP The incidence, associated factors, and predictive nomogram for early death in stage IV colorectal cancer. Int J Colorectal Dis (2019) 34(7):1189–201. 10.1007/s00384-019-03306-1 31089875

[16] Xu Y Xu G Wu H Lin F Mao M Baklaushev VP The Nomogram for Early Death in Patients with Bone and Soft Tissue Tumors. J Cancer (2020) 11(18):5359–70. 10.7150/jca.46152 PMC7391186 32742482

[17] Zhu L Sun X Bai W Nomograms for Predicting Cancer-Specific and Overall Survival Among Patients With Endometrial Carcinoma: A SEER Based Study. Front Oncol (2020) 10:269. 10.3389/fonc.2020.00269 32266128 PMC7096479

[18] Xie G Wang R Shang L Qi C Yang L Huang L Calculating the overall survival probability in patients with cervical cancer: a nomogram and decision curve analysis-based study. BMC Cancer (2020) 20(1):833. 10.1186/s12885-020-07349-4 32873257 PMC7466454

[19] Yokoi A Matsuzaki J Yamamoto Y Tate K Yoneoka Y Shimizu H Serum microRNA profile enables preoperative diagnosis of uterine leiomyosarcoma. Cancer Sci (2019) 110(12):3718–26. 10.1111/cas.14215 PMC6890430 31599471

[20] Lange SS Novetsky AP Powell MA Recent advances in the treatment of sarcomas in gynecology. Discov Med (2014) 18(98):133–40. 25227754

[21] Zhang YY Li Y Qin M Cai Y Jin Y Pan LY High-grade endometrial stromal sarcoma: a retrospective study of factors influencing prognosis. Cancer Manag Res (2019) 11:831–7. 10.2147/CMAR.S187849 PMC6340498 30697075

[22] Penumarthy NL Goldsby RE Shiboski SC Wustrack R Murphy P Winestone LE Insurance impacts survival for children, adolescents, and young adults with bone and soft tissue sarcomas. Cancer Med (2020) 9(3):951–8. 10.1002/cam4.2739 PMC6997066 31838786

[23] Selenica P Conlon N Gonzalez C Frosina D Jungbluth AA Beets-Tan RGH Genomic Profiling Aids Classification of Diagnostically Challenging Uterine Mesenchymal Tumors With Myomelanocytic Differentiation. Am J Surg Pathol (2020). 10.1097/PAS.0000000000001572 PMC8276853 32889887

