# Supplementary material for: A Novel Tool to Predict Early Death in Uterine Sarcoma Patients: A Surveillance, Epidemiology, and End Results-Based Study
Source: Front Oncol. 2020 Nov 26;10:608548. doi: 10.3389/fonc.2020.608548 (PMC7725908; doi:10.3389/fonc.2020.608548)
Supplement: Supplementary file 1 [file DataSheet_1.docx]

Supplementary Material

## Supplementary Figures


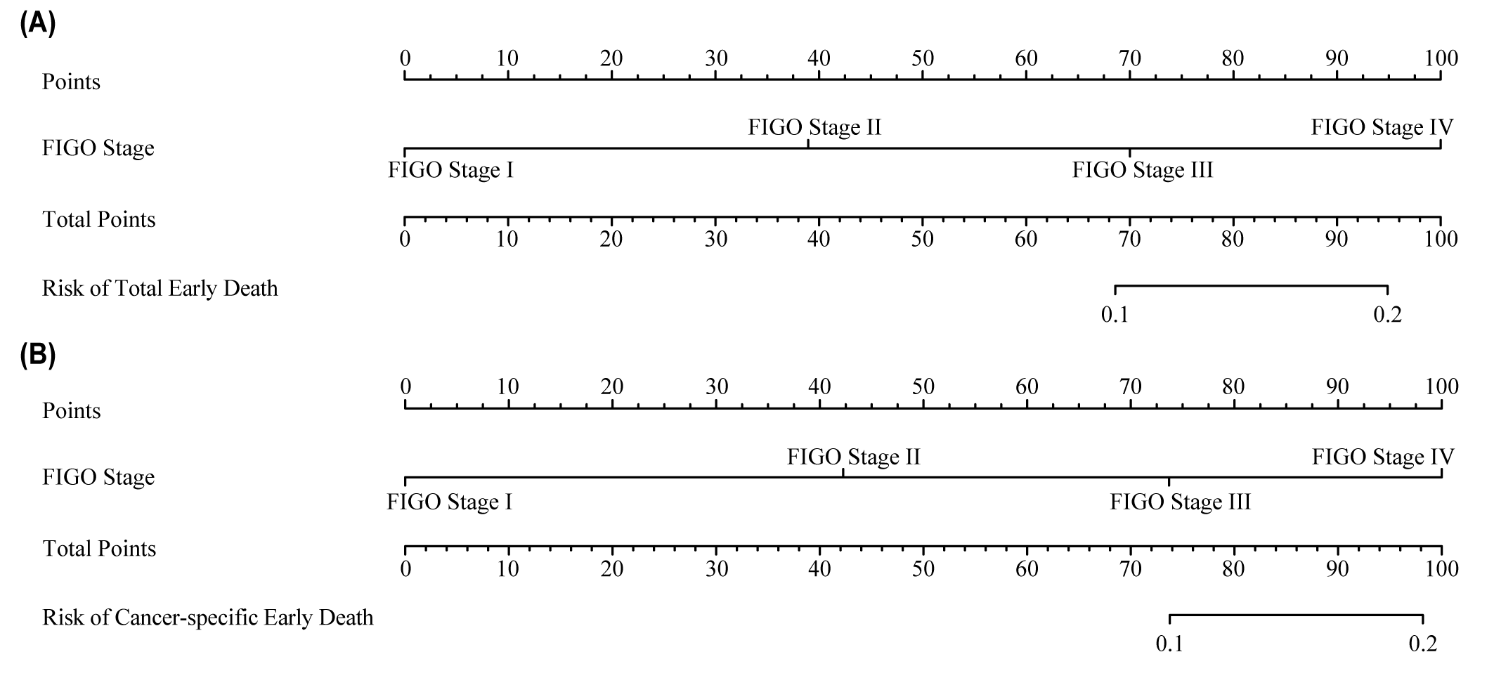


**Supplementary Figure 1.** The International Federation of Gynecology and Obstetrics stages assessing early death of patients with uterine sarcoma. (**A**) The total early death; (**B**) The cancer-specific early death.
